# Molecular investigation of malaria-infected patients in Djibouti city (2018–2021)

**DOI:** 10.1186/s12936-023-04546-x

**Published:** 2023-05-03

**Authors:** Rahma Abdi Moussa, Nasserdine Papa Mze, Houssein Yonis Arreh, Aicha Abdillahi Hamoud, Kahiya Mohamed Alaleh, Abdoul-Razak Yonis Omar, Warsama Osman Abdi, Samatar Kayad Guelleh, Abdoul-Ilah Ahmed Abdi, Mohamed Houmed Aboubaker, Leonardo K. Basco, Bouh Abdi Khaireh, Hervé Bogreau

**Affiliations:** 1 Université d’Aix Marseille, IRD, AP-HM, SSA, VITROME, Marseille, France; 2 IHU-Méditerranée Infection, Marseille, France; 3 Laboratoire de Diagnostic, Caisse Nationale de Sécurité Sociale (CNSS), Djibouti, Republic of Djibouti; 4 Laboratoire National de Référence, Hôpital Général Peltier, Ministère de La Santé, Djibouti, Republic of Djibouti; 5 Laboratoire de Diagnostic, Centre de Santé Communautaire d’Einguela, Ministère de La Santé, Djibouti, Republic of Djibouti; 6 Caisse Nationale de Sécurité Sociale (CNSS), Djibouti, Republic of Djibouti; 7 Programme National de Lutte Contre Le Paludisme, Direction des Programmes de Santé Prioritaires, Ministère de La Santé, Djibouti, Republic of Djibouti; 8 Service de Santé des Armées, Présidence de la République, Djibouti, Republic of Djibouti; 9 UNDP Djibouti, Global Fund to Fight AIDS-TB-Malaria, Djibouti, Republic of Djibouti; 10 Unité Parasitologie et Entomologie, Département Microbiologie et Maladies Infectieuses, Institut de Recherche Biomédicale des Armées, Marseille, France

**Keywords:** Malaria, *Plasmodium falciparum*, *Plasmodium vivax*, Djibouti, Epidemiology, PCR, Rapid diagnostic test

## Abstract

**Background:**

The Republic of Djibouti is a malaria endemic country that was in pre-elimination phase in 2006–2012. From 2013, however, malaria has re-emerged in the country, and its prevalence has been increasing every year. Given the co-circulation of several infectious agents in the country, the assessment of malaria infection based on microscopy or histidine-rich protein 2 (HRP2)-based rapid diagnostic tests (RDT) has shown its limitations. This study, therefore, aimed to assess the prevalence of malaria among febrile patients in Djibouti city using more robust molecular tools.

**Methods:**

All suspected malaria cases reported to be microscopy-positive were randomly sampled (n = 1113) and included in four health structures in Djibouti city over a 4-year period (2018–2021), mainly during the malaria transmission season (January–May). Socio-demographic information was collected, and RDT was performed in most of the included patients. The diagnosis was confirmed by species-specific nested polymerase chain reaction (PCR). Data were analysed using Fisher’s exact test and kappa statistics.

**Results:**

In total, 1113 patients with suspected malaria and available blood samples were included. PCR confirmed that 788/1113 (70.8%) were positive for malaria. Among PCR-positive samples, 656 (83.2%) were due to *Plasmodium falciparum*, 88 (11.2%) *Plasmodium vivax*, and 44 (5.6%) *P. falciparum*/*P. vivax* mixed infections. In 2020, *P. falciparum* infections were confirmed by PCR in 50% (144/288) of negative RDTs. After the change of RDT in 2021, this percentage decreased to 17%. False negative RDT results were found more frequently (*P* < 0.05) in four districts of Djibouti city (Balbala, Quartier 7, Quartier 6, and Arhiba). Malaria occurred less frequently in regular bed net users than in non-users (odds ratio [OR]: 0.62, 95% confidence interval [CI]: 0.42–0.92).

**Conclusions:**

The present study confirmed the high prevalence of falciparum malaria and, to a lesser extent, vivax malaria. Nevertheless, 29% of suspected malaria cases were misdiagnosed by microscopy and/or RDT. There is a need to strengthen the capacity for diagnosis by microscopy and to evaluate the possible role of *P. falciparum hrp2* gene deletion, which leads to false negative cases of *P. falciparum*.

**Supplementary Information:**

The online version contains supplementary material available at 10.1186/s12936-023-04546-x.

## Background

In 2020, the World Health Organization (WHO) reported 241 million cases and 627,000 deaths due to malaria worldwide despite the fact that considerable efforts have been made to control and/or eliminate malaria [1]. Africa is the most affected region, with 95% of malaria cases and deaths occurring on the continent. Malaria still remains a major public health problem in most African countries.

Djibouti is a country in the Horn of Africa with a population of about one million. Its geostrategic position at the entrance to the Red Sea has enabled the country to develop its seaports. Today, Djibouti's port sector is among the most modern in East Africa [2]. Ethiopia, a neighbouring country, is one of the most populous countries in Africa where malaria is known to be endemic [3]. Indeed, Djibouti's seaport is the main maritime gateway to Ethiopia and its market of 100 million people [4].

Malaria was reported for the first time in Djibouti by a French doctor in 1901 [5]. Historically, malaria in Djibouti had been hypo-endemic with unstable transmission. Between 1978 and 1989, the prevalence of malaria in Djibouti increased due to imported cases from neighbouring countries, in particular from Ethiopia [6]. Moreover, malaria outbreaks occurred in 1991 and 1993, followed by the last recorded epidemic in 1999 associated with the expansion of a few strains that were already prevalent [7]. The increasing public health importance of malaria in the country resulted in the creation of National Malaria Control Programme (NMCP) in 2006, the change of first-line anti-malarial treatment from chloroquine to artemisinin-based combination therapy (ACT) in 2006, and the introduction of long-lasting insecticidal mosquito nets (LLINs) in 2006–2011, as well as the massive deployment and use of rapid diagnostic tests (RDT) for malaria [8].

As a result of the efforts of the government to implement malaria control interventions during the pre-elimination period from 2006 to 2012, Djibouti attained an extremely low level of malaria transmission, with only 24 cases recorded in 2012 [8]. Several epidemiological studies carried out during that period of sharp decline in malaria prevalence in Djibouti support this observation [9–12]. As a consequence, Djibouti had set out a pre-elimination plan with the objective to eliminate malaria throughout the country by 2020 [8]. At the time when the pre-elimination programme for the period from 2017 to 2020 was drawn up in 2012, *Plasmodium falciparum* was the predominant malaria species, and infections due to *Plasmodium vivax* represented only about 3% of the total malaria cases.

During the early years of the pre-elimination period, a resurgence of morbidity was noted since 2013 in Djibouti [8]. Moreover, malaria prevalence continued to increase gradually every year. By 2019, the number of malaria cases attained nearly 50,000. This situation alarmed the WHO, which declared a public health emergency in Djibouti. However, the epidemic continued to worsen, reaching over 73,000 cases in 2020. At the same time, the proportion of *P. vivax* cases, based on RDT, increased to 36.7% in 2017 compared to only 3% in 2012 [8]. The sudden increase in malaria prevalence during the last decade in Djibouti can be explained by at least two factors: the introduction of a new mosquito vector and the circulation of *P. falciparum* strains undetectable by RDT.

During the pre-elimination period (2006–2012) in Djibouti, only *Anopheles arabiensis* was the main vector for malaria transmission [8]. The introduction and establishment of a new vector, *Anopheles stephensi*, were first reported in 2012 [13, 14]. Once known as an ‘Asian’ vector, this highly competent *Anopheles* mosquito is now present throughout the Horn of Africa [15–17]. *Anopheles stephensi* thrives in urban areas and feeds at dusk, unlike other *Anopheles* vectors, which prefer to bite late at night [16, 17]. In fact, *An. stephensi* has replaced *An. arabiensis* and has become the predominant vector of malaria in Djibouti. In addition, a recent study also revealed resistance to most commonly used classes of insecticides, notably the pyrethroids [3].

The WHO recommends that all suspected malaria cases be diagnosed by either a RDT or microscopy prior to treatment with ACT to reduce the risk of the progression of the disease towards severe and complicated malaria and the selection and spread of drug-resistant parasites [18]. A preliminary study suggested that a large number (83.5%) of *P. falciparum* strains may be undetectable by histidine-rich protein 2 (HRP2)-based RDT in Djibouti [19]. Although light microscopy is the gold standard for malaria diagnosis, according to the WHO, experienced microscopists are rare in many areas in Africa [20]. This situation has led to a widespread use of HRP2-based RDT at all levels of health service in Djibouti. At present, the country is confronted with a pressing challenge and need for an appropriate tool for malaria diagnosis and surveillance. The extent of misdiagnosis of malaria and its consequences on the ‘real’ epidemiological situation of malaria at present are yet to be assessed. In this context, the use of molecular diagnostic tools has become necessary for a better understanding of the current malaria situation in Djibouti. The objective of the present study was to assess the malaria infection in Djibouti during the epidemic period of 2018–2021 by using nested PCR to understand the dynamics of malaria transmission in the country. In the context of Djibouti being a busy maritime crossroads and part of the Common Market for Eastern and Southern Africa (COMESA), this could be a factor in the spread of malaria not only in the Horn of Africa, but also to the rest of Africa.

## Methods

### Study area

The Republic of Djibouti is a small country (23,000 km^2^) in the Horn of Africa bordered by Eritrea to the north, Ethiopia to the west, Somalia to the south and the Red Sea to the east. Its strategic position and access to the sea at the crossroads of maritime highways, consolidated by modern port infrastructures, make Djibouti a strategic transit point for goods and people between the interior of the African continent and the Middle East and even Asia [21]. In addition, refugees from war-torn neighbouring countries constitute about 3% of the total population of about one million. Two thirds of this highly urbanized population is concentrated in the capital, Djibouti city [22].

The climate is hot and arid with a maximum temperature that varies between 27 and 43 °C. The hot, dry season from May to September is characterized by hot and dry wind (called “Khamsine” in Arabic language) which contributes to high ambient temperature. The ‘cold’ rainy season runs from October to March [23], during which stagnant water is present for long periods in the capital city and larval habitats are created and maintained.

**Fig. 1:**
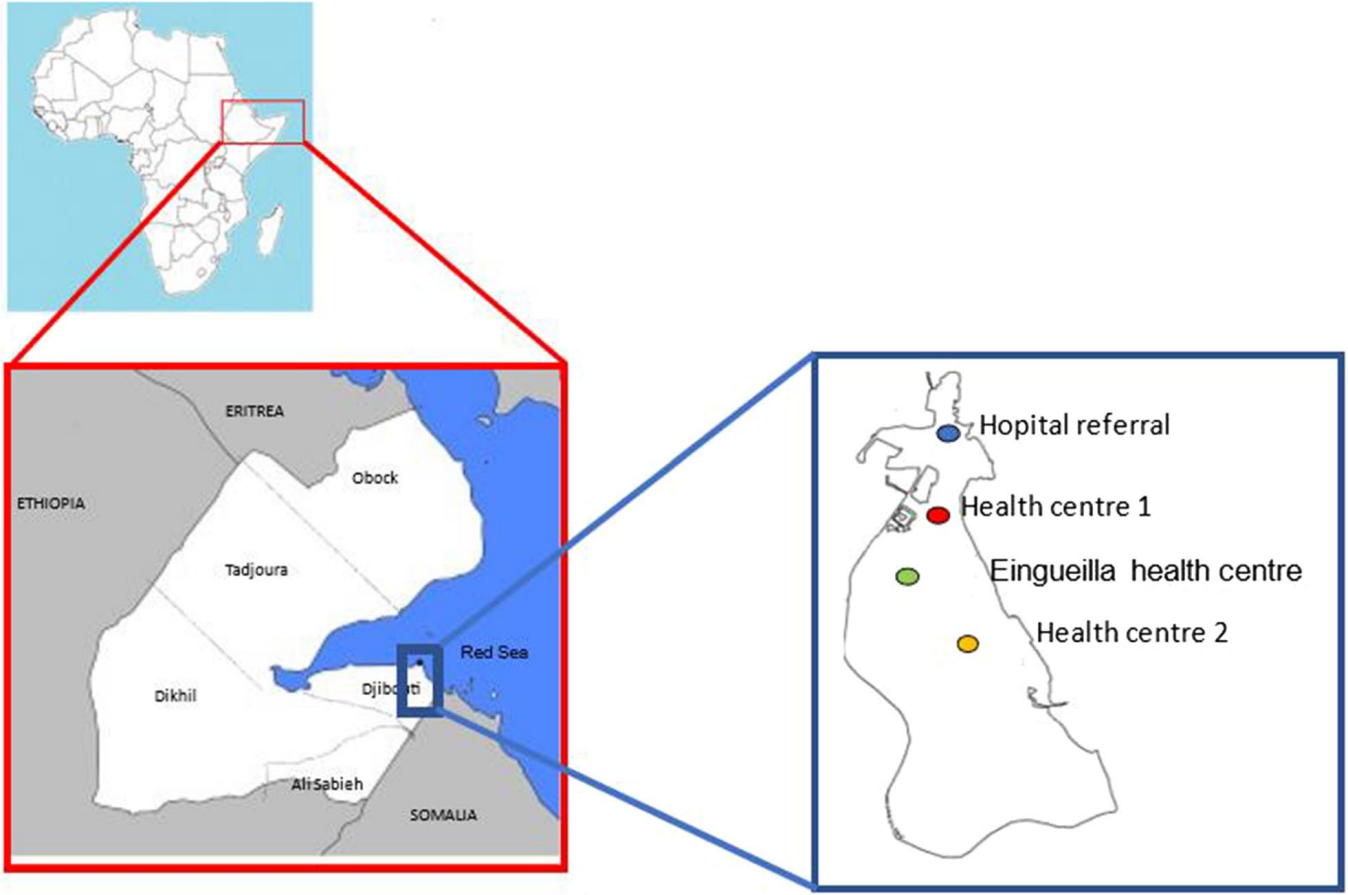
The location of five regions of the country (Djibouti city, Obock, Tadjoura, Dikhil and Ali Sabieh) and four recruitment sites in Djibouti city is shown

The present longitudinal study was carried out in different districts of Djibouti city over a four-year period, from 2018 to 2021, mainly during the malaria transmission season (January-May). For the collection of blood samples, four health structures in Djibouti city were selected on the basis of a high number of outpatients from different districts: health centre 1, health centre 2, Eingueilla health centre, and a referral hospital (Hôpital Général de Peltier) (Fig. 1).

### Inclusion criteria and sample collection

**Fig. 2:**
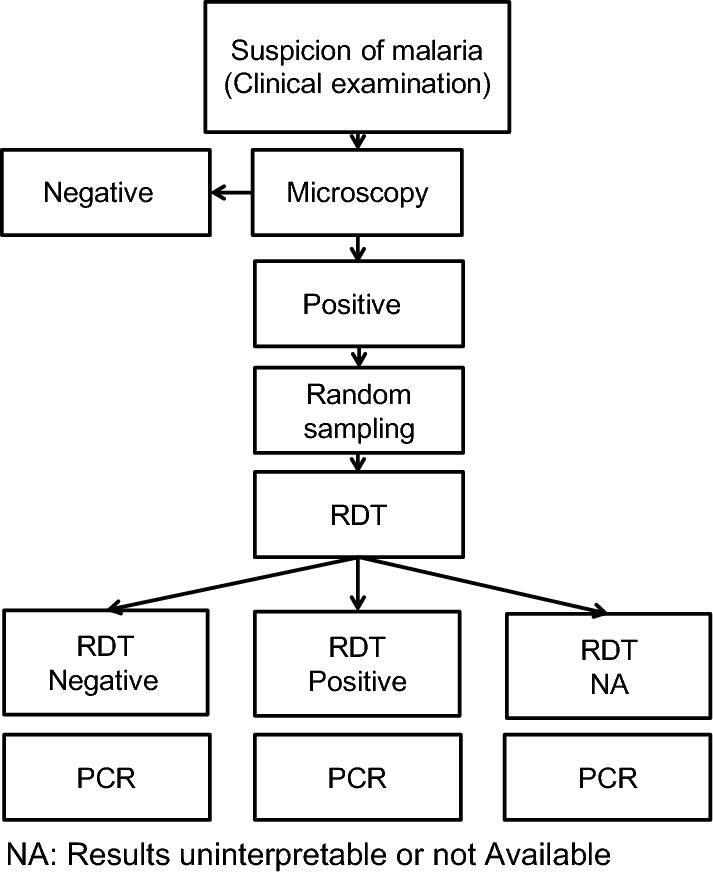
Flow chart describing patient inclusion

The half-day inclusion periods were alternated among four centres according to a pre-established rotation. The inclusion criteria included patients of all ages presenting spontaneously at one of the inclusion health centres or hospital with fever and non-specific signs and symptoms frequently associated with malaria (headache, arthralgia, myalgia, asthenia, loss of appetite, respiratory and gastrointestinal disorders, jaundice). The suspicion of malaria was confirmed initially by microscopic examination of thick blood films, and only patients whose thick film was reported to be positive were randomly selected for inclusion (Fig. 2). After inclusion in the study, RDT was performed depending on the technical capacity of each inclusion centre. All patients reported to have a positive thick film were included in the study regardless of the result of RDT, including some patients with positive microscopy result but without RDT result (due to limited technical capacity of some inclusion centres) because a large majority of Djiboutian *P. falciparum* strains were reported to be undetectable by HRP2-based RDT [19].

After obtaining informed consent from the adult patient or parents (or legal guardians) of children, several drops (approximately 200 µL) of finger-pricked capillary blood were imbibed onto Whatman 3MM filter paper, dried, and stored at − 20 °C in a sealed plastic bag with a dessicant for molecular analysis. Socio-demographic, epidemiological (including the use of bed nets), and mobility data were collected from individual adult patients or parents (or legal guardians) of febrile children. The use of bed nets was classified as “regular” (defined as sleeping under bed nets seven nights a week), “occasional” (defined as sleeping under bed nets 1–6 nights a week), or “never.” The National Reference Laboratory anonymized clinical records, samples, blood slides, RDT, and other identification data of patients in order to preserve privacy and confidentiality of the participants and provided this declaration alongside the agreement for transfer of samples to the Institut Hospitalo-Universitaire – Méditerranée Infection in Marseille, France.

### Rapid diagnostic tests

Two different brands of RDT were used in this study. These RDTs were provided by the Djiboutian Ministry of Health free of charge to the health centres and referral hospital where the present study was conducted. CareStart Malaria Pf/Pv (HRP2/pLDH) Ag Combo RDT (ACCESS BIO Somerset, NJ) was used in 2018–2020. This RDT detects *P. falciparum*-specific HRP2 and *P. vivax*-specific lactate dehydrogenase (Pv-pLDH). The poor performance of this RDT in the field in Djibouti led to its replacement by a new RDT. The Biosynex® malaria P.f/Pan test (Biosyntex; Illkirch-Graffenstaden, France) was used in 2021. It detects PfHRP2 and *Plasmodium* genus-specific (all human *Plasmodium* spp.) lactate dehydrogenase (pLDH pan). Both tests were performed according to the manufacturers' instructions.

### Microscopy

Thin smears were fixed with pure methanol, and thick and thin smears were stained with 10% Giemsa for 20 min. After drying the slides, the smears were examined with a light microscope under oil immersion at a magnification of 1000×. Thick smears were examined to confirm RDT results. A thick smear was considered negative if no asexual parasites of *Plasmodium* were observed after an examination of 200 fields. Parasitaemia was not determined.

### DNA extraction

Parasite DNA was extracted from blood of patients collected on RDT or spotted on Whatman™ FTA® cards (GE Healthcare). RDT cassettes were dismantled, and the area of the nitrocellulose membrane where DNA is most concentrated was cut out. DNA in lysed blood on nitrocellulose membrane was extracted as described by Cnops et al. [24]. Three disks (1-mm diameter) were punched out from filter paper saturated with blood sample and transferred to 96-well plate. Parasite DNA extraction from dried blood spots and nitrocellulose membrane was performed using an automated magnetic bead-based robot (MagMAX™-Express, Thermo Fisher Scientific, Montigny-le-Bretonneux, France) following the manufacturer's instructions.

### Polymerase chain reaction (PCR)

All available blood samples (n = 1113) from microscopy-positive, RDT-positive or RDT-negative patients were analysed by PCR. *Plasmodium* species was identified using nested PCR protocol developed by Snounou et al. [25] and modified using fluorescent labelled oligonucleotides as previously described [26]. The PCR products were analysed by capillary electrophoresis using Applied Biosystems™ 3130XL 16-capillary array genetic analyzer (Thermo Fisher Scientific, Illkirch, France). The primers used to detect four human *Plasmodium* species are shown in Additional file 1.

### Statistical analysis

Data were entered into an Excel spreadsheet (Microsoft Office Excel 2011; Redmond, WA). Fisher's exact test was used to compare proportions between categorical variables. Cohen's kappa coefficient was calculated to estimate the degree of agreement between the results of PCR and RDT. All statistical tests were performed using R version 4.1.2 [27]. The interpretation of kappa coefficient was as follows: < 0, no agreement, 0.01–0.20, slight agreement, 0.21–0.40 fair agreement, 0.41–0.60 moderate agreement, 0.61–0.80 substantial agreement and 0.81–1.0 almost perfect agreement [28].

## Results

### Patient characteristics

A total of 1113 patients with samples available for molecular diagnosis were included during the malaria transmission season in 2018–2021 (number of samples [n] = 80 in 2018, 187 in 2019, 341 in 2020, and 505 in 2021). The samples were collected from Health Centre 1 (n = 615), Health Centre 2 (n = 325), Einguella Health Centre (n = 29), and Djibouti City General Hospital (n = 144). The proportion of males (61.1%, 520/851) was significantly higher (*P* < 0.05, 95% confidence interval [CI], 57.7–64.4%) than females (38.9%, 331/851), with a sex-ratio of 1.6 (262 missing data on sex). The mean (± standard deviation [SD]) and median ages were 30.4 years (± 18.0 years) and 28 years, respectively, ranging from 2 months to 95 years.

### Malaria infection

PCR was successfully performed and interpretable in all 1113 (100%) samples, consisting of 729 dried blood spots and 384 RDTs, available for molecular diagnosis. The overall prevalence of malaria based on PCR-confirmed cases was 70.8% (788/1113) in 2018–2021. This prevalence was 81.2% (65/80) in 2018 between September and December and 64.2% (120/187) over the whole of 2019. In the period September-December only, the prevalence in 2019 was 76.1% (54/71) and the difference with the previous year was not statistically significant (*P*-value = 0.55). Between 2020 and 2021, the prevalence in the period January-April decreased significantly (*P*-value = 0.02) from 75.9% (259/341) to 68.1% (344/505).

Of the PCR-confirmed malaria-infected patients (n = 788), 656/788 (83.2%) were due to *P. falciparum*, 88/788 (11.2%) were due to *P. vivax*, and 44/788 (5.6%) were due to mixed *P. falciparum/P. vivax* infections (Table 1). *Plasmodium ovale* and *Plasmodium malariae* were not found in this study. Considering both mono- and mixed infections, the proportions of PCR-confirmed *P. falciparum* malaria cases were 70% in 2018, 51.3% in 2019, 69.5% in 2020, and 61.6% in 2021. For *P. vivax*, the corresponding proportions were 18.8%, 19.3%, 9.7%, and 9.5% (Additional file 1: Data S1).

**Table 1:** Malaria diagnosis by PCR in patients with suspected malaria consulting three health centres and a referral hospital in Djibouti city in 2018–2021

Year	All PCR-confirmed samples		*P. falciparum*		*P. falciparum-P. vivax*		*P. vivax*	
	N	%^a^	n	%^b^	n	%^b^	n	%^b^
2018	65	81.2	50	76.9	6	9.2	9	13.9
2019	120	64.2	84	70.0	12	10.0	24	20.0
2020	259	75.9	226	87.3	11	4.2	22	8.5
2021	344	68.1	296	86.0	15	4.4	33	9.6
Total	788	70.8	656	83.2	44	5.6	88	11.2

N, Total number of samples; n, number of samples for each *Plasmodium* spp. or mixed infections. ^a^Percentage among all samples tested by PCR by year, ^b^percentage among all PCR-confirmed infected samples by year

The mean (± SD) age of patients with symptomatic malaria infection confirmed by PCR was 30.5 (± 18.2) years. There was no significant difference with the mean age of the patients with PCR-negative results included in the study (30.9 ± 19.3 years; *P* = 0.7). The proportions of *P. falciparum* infections confirmed by PCR in 3 different age groups (i.e., under 18 years, 18–34 years, and over 34 years) did not differ significantly (*P* = 0.07) (Table 2). Considering a narrower age range of one decade, there was a lower number (*P* < 0.05) of PCR-confirmed malaria-infected patients among 0–9 year olds and 60–79 year olds. On the other hand, patients in the 10–29 year old age group were proportionally overrepresented (odds ratio [OR] = 1.6; 95% CI: 1.16–2.24, *P* = 0.003, Additional file 2: Fig. S1).

**Table 2:** Age groups of symptomatic, PCR-confirmed malaria-infected patients in Djibouti city in 2019–2021

Year	Age groups							
	< 18 years		19–34 years		> 34 years		Total	
	n	%	n	%	n	%	N	%
*PCR-negative*								
2019	2	12	9	53	6	35	17	7
2020	12	15	28	36	38	49	78	33
2021	59	43	25	18	54	39	138	59
Total	73	31	62	27	98	42	233	100
*PCR-positive*								
2019	9	16	34	61	13	23	56	9
2020	32	13	92	37	124	50	248	42
2021	121	42	81	28	85	30	287	49
Total	162	27	207	35	222	38	591	100

N, Total number of samples; n, number of samples in each age group. Age of patients was not recorded in 2018

Concerning the sex of the patients, of the 851 patients with a PCR result and their sex documented, 75.4% (392/520) of males and 64.7% (214/331) of females had malaria. The OR of malaria prevalence among males was 1.67 (95% CI: 1.22–2.29, *P* < 0.001) times higher than in females.

### Association between bed net use and malaria infection

Information on the reported use of bed net was obtained from 600 of 1113 (53.9%) febrile patients. Of these respondents, 64.2% (n = 385) reported that they do not possess a bed net or they have at least one bed net in the household but never use it. Only 215 of 600 (35.8%) reported to sleep under a bed net, of whom 34 (5.7%) were occasional users and 181 (30.2%) were regular users. The proportion of malaria infections was 69.9%, 61.8%, and 59.1% among non-users, occasional users, and regular users, respectively (Table 3). The regular use of insecticide-treated bed nets was associated with a decreased likelihood of malaria infection (OR: 0.62, 95% CI: 0.42–0.92, *P* < 0.013), compared to those who did not use bed nets (Table 3).

**Table 3:** Bed net use and malaria infection among febrile patients consulting in health centres and referral hospital of Djibouti city in 2018–2021

Bed net use*	Number of patients	PCR-positive		PCR-negative	
	N	n	%	n	%
Never	385	269	69.9	116	30.1
Occasional	34	21	61.8	13	38.2
Regular*	181	107	59.1	74	40.9
Total	600	397		203	

N, total number of patients who provided information on bed net use and whose diagnosis was confirmed by PCR; n, number of PCR-positive or PCR-negative patients. *Significant statistical association between bed net use and malaria infection with Fisher exact test: Odds-ratio_(Never vs Regular)_ 0.62 (0.42–0.92), *P*-value _(Never vs Regular)_ = 0.013

### Performance of RDT for malaria diagnosis

RDTs detected *P. falciparum* in 39.0% (369/946) of the samples tested. During the 4-year period of the study, the percentages of RDT-positive *P. falciparum* were 84.6% in 2018, 17.3% in 2019, 23.0% in 2020, and 53.5% in 2021 (Additional file 1: Data S1). Over the entire study period, only 318 of 595 (53.4%) *P. falciparum* infections confirmed by PCR were RDT-positive for *P. falciparum*. The performance of RDTs for the detection of *P. falciparum* is detailed by year in Table 4.

**Table 4:** Performance of rapid diagnostic tests for malaria diagnosis in Djibouti city, 2018–2021

Performance	Year^1^							
	2018		2019		2020		2021	
	n	%	n	%	n	%	n	%
*P. falciparum*								
True positive	55	71	27	14	62	22	174	47***
False positive	11	14	6	3	6	2	25	6.7*
True negative	12	15	85	45	76	26	111	29
False negative	0	0	69	37	144	50	64	17***
Total	78	100	187	100	288	100	374	100
Sensitivity		100		28.1		30.1		73.1***
Specificity		52.2		93.4		92.7		81.6*
PPV		83.3		81.8		91.2		87.7
NPV		100		55.2		34.6		63.4
Accuracy		85.9		59.9		47.9		76.5
*P. vivax*								
True positive	9	12	27	14	23	8	23	6*
False positive	1	1	4	2	3	1	67	17.9***
True negative	62	79	147	79	254	88	272	73
False negative	6	8	9	5	8	3	12	3
Total	78	100	187	100	288	100	374	100
Sensitivity		60.0		75.0		74.2		65.7
Specificity		98.4		97.3		98.8		80.2***
PPV		90.0		87.1		92.0		51.1
NPV		98.4		94.2		98.4		97.1
Accuracy		97.3		93.0		97.9		90.8

^1^In 2018–2020, CareStart malaria Pf/Pv HRP2/pLDH RDT was used. In 2021, Biosynex malaria P.f/pan pLDH was used. *P*-values for the comparison between Biosynex_2021_ vs CareStart RDT_2018-2020_: < 0.01*, < 0.001**, < 0.0001***. PPV: Positive predictive value; NPV: Negative predictive value

The proportion of false-negative RDTs based on PCR results as the reference was 0% in 2018 but reached 50.0% (144/288) in 2020 with CareStart® HRP2-based RDT. However, 17.1% of false negatives (64/374) were observed in 2021 with Biosynex RDT (pf-HRP2/pan/LDH). In contrast, the proportion of false-positive CareStart® RDTs was 14% (11/78), 3% (6/187) and 2% (6/288) in 2018, 2019, and 2020, respectively. In 2021, 6.7% (25/374) of Biosynex® RDTs were false positives (*P*_2020 vs 2021_ = 0.05) (Table 4).

The sensitivity of CareStart® RDT in 2018–2020, compared to PCR, for the detection of *P. falciparum* was very high in 2018 with 100% (95% CI, 93.4–100%), but the specificity was low (52.2%; 95% CI, 30.6–73.2%). However, in 2019, the sensitivity of CareStart® RDT decreased to 28.1% (95% CI, 22.6–35.5%). The sensitivity and specificity of Biosynex® RDT in 2021 was 73.1% (95% CI, 67.5–79.0%) and 81.6% (95% CI, 74.1–87.7%), respectively. The difference in the sensitivity (*P* < 0.0001) and the specificity (*P* < 0.01) in the detection of *P. falciparum* with two RDTs was statistically significant.

As for *P. vivax* infections, the proportion of false-negative RDTs compared to PCR as the reference method was low throughout the study period with the maximum of 4.8% (9/187) in 2019. By contrast, a high proportion of false positives (17.9%, 67/374) was observed in 2021. Of these false positives, 45 (67.2%) mixed infections detected by Biosynex® RDT showed only *P. falciparum* by PCR. The sensitivity of RDTs to detect *P. vivax* ranged from 60 to 75% depending on the year (Table 4). The sensitivity to detect *P. vivax* with the two RDTs did not show any statistical difference (*P* = 0.3), but a significant difference was observed in the specificity of the two RDTs (*P* < 0.0001). PCR and RDT results showed poor agreement for both *P. falciparum* and *P. vivax* (kappa coefficient, 0.28; 95% CI, 0.21–0.40).

### Distribution of false-negative RDT results in different districts of Djibouti city

**Fig. 3:**
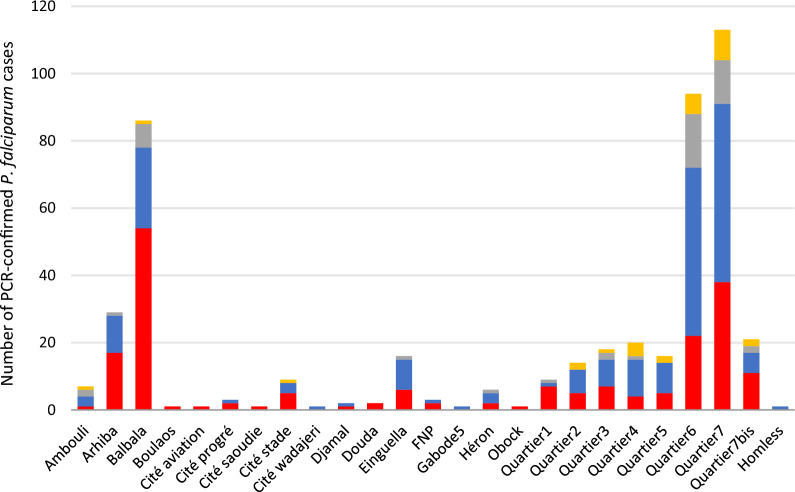
The results of the RDT from PCR-confirmed *P. falciparum* cases: negative (red), P.f. (blue), P.f./P.v.(grey) and P.v. (yellow) are represented according to the residence of the patients. Only patients with PCR-confirmed *P. falciparum* infection and a valid RDT are counted. Patients who did not provide their place of residence (n = 120) are not represented. District FNP: Force Nationale de la Police

For the detection of *P. falciparum*, the proportion of false-negative RDT cases (i.e. PCR-positive but RDT-negative) varied from one district to another (Fig. 2). Balbala district was the most affected by *P. falciparum* strains presumably undetectable by RDT (54/86, 62.8%), followed by Arhiba district (17/29, 58.6%), “Quartier 7” district (38/113, 33.6%), and “Quartier 6” district (22/94, 23.4%). Patients from Balbala district had consulted the referral hospital, health centre 1, or health centre 2. Each of these three health facilities had reported false negative RDTs from this district (Fig. 3).

## Discussion

Over the past decade, Djibouti has been facing a re-emergence of malaria with a worsening of the situation in recent years [8]. In this health crisis, Djibouti is also facing the emergence of other infectious diseases, such as dengue fever, clinical presentation and period of transmission of which are similar to those of malaria [29]. In addition, comprehensive diagnostic tools for infectious diseases are either not available or possibly inappropriate for the country. The widespread use of HRP2-based RDT is hampered by the presence of some Djiboutian *P. falciparum* strains with deletion of the target gene [19]. Moreover, microscopic examination of blood smears requires a high expertise that the country needs to improve. The sensitivity of microscopy remains relatively low and is largely outperformed by PCR [20, 30]. Therefore, the evaluation of malaria prevalence and the ‘real’ malaria burden raises several questions. In this epidemiological context, the present study aimed to address this question using robust molecular tools. The diagnosis of patients with signs and symptoms suggestive of malaria was confirmed by using nested PCR as the reference method. *Plasmodium* species were also determined in order to generate a reliable diagnostic database.

### Malaria prevalence

Despite the limitations of the diagnostic tools available in Djibouti, 71% of suspected symptomatic malaria cases were confirmed by PCR in the present study. This prevalence is higher than that reported by the country’s NMCP in 2019 [8] (i.e. 24% of confirmed suspected malaria cases) or previous study conducted in Ethiopia [31]. Variations between years were observed in the current study. This may be partly due to the sampling period, the extension of the transmission period due to climate change, the occurrence of other epidemics during the study period, or the change of RDT in 2021. As a point of comparison, if the available data in Djibouti city between January and September 2019 provided by the NMCP are considered, 26.8% of suspected malaria cases were confirmed by RDT or microscopy [8]. During the same period, data analysed in the present study have shown that 56.3% of suspected malaria cases were confirmed by PCR. This difference can be partly explained by the more stringent inclusion criteria used in the present study, in which the initial diagnosis of suspected malaria cases was based on clinical examination with additional on-site confirmation by microscopy or RDT, whereas only clinical examination was considered in previous studies [8]. Furthermore, PCR performed in the present study is more sensitive than RDT, as previously shown in other studies [20]. A higher confirmation rate of malaria diagnosis is, therefore, expected in the present study.

In the present study, it was observed that malaria affected all age groups in Djibouti city. The age distribution of the patients included broadly corresponded to the Djiboutian population structure observed in the latest census (Additional file 2: Figure S1). However, selection biases related to the attendance of different health facilities could have occurred. An under-sampling of young children aged 0–9 years among the patients included in this study was observed (11% observed vs 20% expected). To limit this problem, the comparison of malaria-infected and uninfected populations included in the current study can provide relevant epidemiological data on the malaria situation in Djibouti. Even though the mean age was not significantly different between these two groups, three differences were observed (Additional file 2: Figure S1). Firstly, the proportion of young children (0–9 years) was lower among malaria-infected patients than in the population of uninfected patients. Population-based malaria interventions focused on young children could explain this observation. The distribution of LLINs for children under 5 years old could contribute to their under-representation among malaria-infected patients. The latest studies reported a coverage rate of 41% in this young vulnerable population [32]. The high burden of other diseases, such as pneumonia or diarrhoea, could also contribute to more frequent consultations of children among patients without malaria [33]. Secondly, the risk of malaria infection is higher in the age group 10–29 years old (*P* = 0.003). This difference seems to be associated with nocturnal outdoor activities. Indeed, males are more likely to be infected, especially in this age group. This is consistent with the traditional activities of females who tend to stay home more frequently, leading to a lower level of exposure to *Anopheles* mosquitoes [31].

The third statistically significant difference observed between *Plasmodium*-infected and uninfected patients was in the older age groups (> 69 years). The proportion of elderly patients was higher among those who were not infected with malaria (10.3% vs 5.6%, *P* = 0.02). It is unlikely that these differences can be attributed to the acquisition of immunity in older people [34] because these proportions among malaria-infected patients and the general Djiboutian population are similar [35]. Moreover, malaria transmission in Djibouti remains seasonal with insufficient levels to allow the acquisition of immunity, as observed in hyperendemic regions where malaria is stable [34]. Although some immunity may be acquired in areas of intermediate level of transmission [36], the recent aggravation of the malaria situation in Djibouti (within less than one decade) is inconsistent with the differences with slightly younger age groups. Given this situation, it is more likely that the higher proportion of older patients among those who were not malaria infected is related to other conditions. For example, the on-going coronavirus disease-19 (COVID-19) pandemic since 2020 could explain the increase in the number of consultations in this age group [37].

### Bed nets

An appropriate use of LLINs has been shown to result in the reduction of malaria-associated morbidity and mortality, especially among children, pregnant women, and other vulnerable people [38]. However, bed net use depends on the type of household and individual and community knowledge about the usefulness of bed nets in preventing the risk of contracting malaria. A review by the Djiboutian NMCP reported that bed net utilization by the population has been sub-optimal in recent years. The reasons include the following: (i) some households do not use bed nets because they live in narrow premises and do not have a bed where bed nets can be hung, (ii) while others do not know how to use the bed nets properly and wash, dry, and expose them regularly to the sun, which rapidly degrades the impregnated insecticide [39]. Despite these limitations, the present study confirms the effectiveness of LLINs to protect against *Anopheles* bites and limit malaria transmission in regular users. Given the current re-emergence of malaria in Djibouti, it will be important for the country to accelerate their vector control policies with massive distribution of LLINs and maintain a tight follow-up on its utilization, as recommended by the WHO. There is also a need to raise community awareness with health education campaigns focusing on the correct use and maintenance of bed nets to optimize their protective effects [40].

### *Plasmodium* species

In line with earlier studies conducted in Djibouti [6, 10, 11], the present study showed that the majority of malaria infections in symptomatic patients are due to *P. falciparum* (83.3%) in Djibouti city, followed by *P. vivax* (11.2%). No other *Plasmodium* spp. was detected by PCR in the present study in concordance with the results of previous studies [11].

This is the first study that assessed the prevalence of *P. vivax* in Djibouti over a period of 4 years using PCR. The results showed that *P. vivax* accounts for 16.8% of malaria-confirmed cases in Djibouti city, of which 5.6% are mixed *P. falciparum*-*P. vivax* infections. Previous monitoring based on RDT or microscopy reported higher percentages of *P. vivax*. The latest available data from the NMCP reported 13,373 *P. vivax* infections in 2019, which represents 27% of malaria infections [8].

Four factors could explain the lower proportion of *P. vivax* malaria in the present study.

i. The present study was mainly conducted during the period of high malaria transmission in Djibouti. In the last two years (i.e. 2020–2021), very few patients were included during the dry season when malaria transmission decreases sharply. During this period, *P. vivax* became the predominant species, presumably because of relapses, even though its transmission was also greatly reduced [41, 42].
ii. The recent attention of the specificities of *P. vivax* in the management of patients may also have been accompanied by improved effectiveness of malaria control programmes. The use of primaquine allows the elimination of hypnozoites in the liver. It is prescribed for the treatment of *P. vivax*-infected patients implemented in Djibouti since 2019 [39]. Although the 14-day protocol requiring a prior glucose-6-phosphate dehydrogenase (G6PD) assay is restrictive and limits compliance, the initial effects of this specific management may have had an effect on parasite populations.
iii. The regional context may have had an effect on the situation in Djibouti. The Horn of Africa is the region with the highest prevalence of *P. vivax* on the continent [43]. Ethiopia is the second most populous country in Africa [44]. The prevalence of *P. vivax* in the general population of Ethiopia is over 8%, with wide variations between regions [45]. Given the large volume of trade between Ethiopia and Djibouti [46], the evolution of the malaria situation in one of the two countries can have a large impact on its neighbour [47]. A trend towards a decrease in the proportion of malaria infections due to *P. vivax* has been observed in certain regions of Ethiopia, sometimes even leading to a reversal of the trends, with *P. falciparum* becoming predominant in an area where *P. vivax* was previously predominant [48–50].
iv. Finally, the failure of the RDTs used in Djibouti may have produced two effects which are consistent with a higher proportion of *P. vivax* reported in the previous studies [8]. The first immediate effect is an underestimation of *P. falciparum* due to the propagation of strains with deleted *P. falciparum hrp2* (*Pfhrp2*) gene, which will not be detected by HRP2-based RDTs [19]. This diagnostic problem may also have a second effect on the relative proportion of these two plasmodial species by favouring the transmission of *P. falciparum*. Strains without the *Pfhrp2* gene will not be detected or will be detected late (for example, when the parasitaemia increases, facilitating microscopic examination or rendering LDH-based RDT sensitive enough to detect the parasites). This situation offers an intra-species selective advantage to *Pfhrp2*-deleted *P. falciparum* strains, as evidenced by their spread throughout malarious areas [1, 51], but it could also lead to an inter-species selective advantage. *Plasmodium falciparum* strains with deleted *Pfhrp2* gene, which is less detectable by many currently available RDTs, would then have an advantage over the *P. vivax* strains in the face of control strategies based increasingly on RDTs and post-diagnostic treatment only policy [18].

The current study also showed that errors in RDT readings could lead to false positives for *P. vivax.* This problem occurred after the introduction of the new test in 2021. Mixed *P. falciparum-P. vivax* infections had been reported in patients infected with *P. falciparum* only, as confirmed by PCR. In such cases, surveillance based mainly on RDTs will overestimate the proportions of *P. vivax* compared to *P. falciparum*. In contrast, the present study based on PCR avoids this bias.

These different factors could explain the lower prevalence of *P. vivax* in the present study, with a decreasing prevalence observed in the last two years of the study (2020–2021). On the other hand, it is difficult to predict how the importation and establishment of *An. stephensi* in Djibouti could modify the relative transmission of these two plasmodial species. Preliminary data suggested that the arrival of this Asian vector may be accompanied by ecological changes favourable to the spread of *P. vivax* [14]. In this context, the reinforcement of surveillance of plasmodial populations in Djibouti with adapted tools, which is part of the NMCP’s control strategies, is relevant and should be pursued.

### Performance of RDT

Using PCR as the reference method, the present study highlights the shortcomings of malaria diagnosis that relies on HRP2-based RDT. High proportions of false-negative *P. falciparum* were observed during the last three years of the present study. The highest proportion of false-negative *P. falciparum* (50%) was found when diagnosis was established using HRP2-based RDT. This could be explained by several factors, including low parasite density, low target antigen concentration, poor quality of RDTs due to poor stockage conditions or incorrect warehousing, incorrect use of RDTs by technicians or *Pfhrp2* gene deletion [52–55]. Due to the unavailability of experienced local microscopists and alternative coloration methods, the proportion of malaria cases who were false-negative because of low parasitaemia cannot be estimated from the present study. False negativity due to *Pfhrp2* gene deletion is of potentially great concern as a recent study in Djibouti detected false-negative *P. falciparum* by HRP2-based RDT caused by a deletion of the *Pfhrp2* gene [19]. Moreover, other recent studies in the neighbouring countries, Ethiopia and Eritrea, have also demonstrated the problem of non-detection of *P. falciparum* strains by HRP2-based RDT because of *Pfhrp2* gene deletion [56, 57]. Indeed, the prevalence of *Pfhrp2* gene deletion has reached high levels (9.4–83.5%) in the Horn of Africa [19, 56–58].

The distribution of the proportions of false-negative RDTs across Djibouti city suggested a structuring of these strains in certain districts of the city. Balbala, Quartier 6, Quartier 7, and Arhiba were most affected by *P. falciparum* strains undetected by HRP2-based RDT. This is all the more interesting as patients from the same district consulted in different health centres of this study, thus limiting an introduction of a possible bias due to recruitment and practice in different health centres (e.g., storage conditions of RDT or interpretation of RDTs). There may be an oligoclonal expansion of *Pfhrp2*-deleted *P. falciparum* strains, leading to microstructures in Djibouti city as observed in the previous epidemic in 1999 [7]. Further analysis by genotyping *P. falciparum* populations in Djibouti is required to establish the numbers of different circulating strains and characterize their genetic profiles, origins, and evolution in time and place. In addition, a small proportion (2.3%) of false-negative *P. vivax* by RDT was also confirmed by PCR. This result could probably be explained by low parasitaemia [59].

False-positive *P. falciparum* was observed by HRP2-based RDT. The highest proportion of false positives was 14% in 2018 and 7% in 2021. This could be due mainly to the persistence of HRP2 in recently recovered *P. falciparum*-infected patients or those presenting the rheumatoid factor, which cross-reacts with HRP2 [60, 61]. In contrast, significantly high proportions of false positives for *P. vivax* (7%) were mainly due to misinterpretation of the new RDT introduced in one of the health centres. These results may also suggest an effect of sample storage prior to molecular analysis. However, the overall sensitivity and specificity showed poor performance of RDTs used, with sensitivity for detection of *P. falciparum* and *P. vivax* of 58% and 81%, respectively, and specificity of 80% and 97%, respectively. A high rate of misdiagnosis at country level and the accompanying inappropriate anti-malarial drug treatment are potential sources and reasons for ineffective malaria control as well as the spread of drug-resistant *P. falciparum* (and *P. vivax*) strains. Indeed, recent studies have documented ACT treatment failure in Djibouti [62] and the presence of mutations in the *P. falciparum kelch 13 (Pfk13)* gene associated with reduced susceptibility to artemisinins in Eritrea [63]. To regain the momentum that Djibouti had lost about a decade ago for eliminating malaria, an accurate and rapid diagnosis, followed by an effective treatment with ACT, delivered, managed, and followed by properly trained, health care personnel is necessary.

### Limitations of study

There are several limitations in this study. First, due to scarcity of qualified microscopists in the country and lack of compliance to the national diagnostic guidelines, parasitaemia was not available. Parasitaemia plays an important role in RDT detection. Parasite density below the RDT detection threshold or very high parasite density can influence the performance of RDT [64]. Secondly, samples were collected only in Djibouti city. Therefore, the data generated in this study do not reflect malaria prevalence in the entire territory of Djibouti. An earlier seroprevalence study showed that the prevalence of *P. falciparum* was high in two other districts of Djibouti [11]. Third, the study population represents part of the patient population randomly selected in a limited number of health centres and one referral hospital in Djibouti city. Fourth, the malaria situation in the general population was not explored in the present study. Further studies need to be conducted, not only in other parts of the country, but also in randomly selected households, to determine the true prevalence of malaria in the general population in Djibouti. Fifth, the poor performance of two RDTs approved by the Djiboutian health authorities was unexpected, in particular the poor capacity of one of the RDTs to detect *P. falciparum* with pLDH pan. The issue of sensitivity and specificity of RDTs needs to be further investigated on a wider scale in the country. Given the rapidly changing malaria situation and the latest WHO recommendations, a new RDT RapiGEN Biocredit Pf/Pv (pLDH/pLDH), subsidized by the Global Fund, is currently being deployed in 26 health centres in Djibouti and requires further evaluation to assess and adjust malaria control interventions in the country.

## Conclusions

PCR verification of suspected malaria cases in Djibouti city suggested that 29% of non-malarial cases were misdiagnosed by microscopy and/or RDT and mistakenly treated for malaria. The predominance of *P. falciparum* is confirmed, and *P. vivax,* to a lesser extent, remains a public health concern. The performance of HRP2-based RDT to detect *P. falciparum* was poor and led to its successive changes in February 2021 to pLDH-based RDTs. It is imperative to evaluate regularly the performance of RDTs in Djibouti as there are few well-trained microscopists in the country and false-negative RDTs lead to inadequate or inappropriate treatment and increased malaria mortality and morbidity. Further molecular studies are required to characterize *P. falciparum* populations that yield false-negative HRP2-based RDT results in order to understand their distribution, origin, and dynamics at both country and regional level.

## Supplementary Information

**Additional file 1: Data S1.** Number and percentage of positive samples for Plasmodium falciparum and Plasmodium vivax by RDT and PCR in Djibouti city, 2018–2021.

**Additional file 2: Figure S1.** Age of patients by malaria status from four health facilities in Djibouti city between 2019 and 2021.

## Data Availability

All data generated or analysed during this study are included in this published article and its additional information files.

## Abbreviations

ACT: Artemisinin-based combination therapy
COMESA: Common Market for Eastern and Southern Africa
COVID-19: Coronavirus disease-19
HRP2: Histidine-rich protein 2
LLIN: Long-lasting insecticide-treated mosquito nets
NMCP: National Malaria Control Programme
NPV: Negative predictive value
OR: Odds-ratio
PCR: Polymerase chain reaction
pLDH: Plasmodial lactate dehydrogenase
pLDH pan: *Plasmodium* genus-specific lactate dehydrogenase
PPV: Positive predictive value
*Pfhrp2*: *P. falciparum hrp2* gene
*Pfk13*: *P. falciparum kelch 13* gene
Pv-pLDH: *Plasmodium vivax*-specific lactate dehydrogenase
RDT: Rapid diagnostic test
SD: Standard deviation
WHO: World Health Organization
