# Correction: Molecular investigation of malaria-infected patients in Djibouti city (2018–2021)

**DOI:** 10.1186/s12936-023-04598-z

**Published:** 2023-05-30

**Authors:** Rahma Abdi Moussa, Nasserdine Papa Mze, Houssein Yonis Arreh, Aicha Abdillahi Hamoud, Kahiya Mohamed Alaleh, Abdoul-Razak Yonis Omar, Warsama Osman Abdi, Samatar Kayad Guelleh, Abdoul-Ilah Ahmed Abdi, Mohamed Houmed Aboubaker, Leonardo K. Basco, Bouh Abdi Khaireh, Hervé Bogreau

**Affiliations:** 1 Université d’Aix Marseille, IRD, AP-HM, SSA, VITROME, Marseille, France; 2 IHU-Méditerranée Infection, Marseille, France; 3 Laboratoire de Diagnostic, Caisse Nationale de Sécurité Sociale (CNSS), Djibouti, Republic of Djibouti; 4 Laboratoire National de Référence, Hôpital Général Peltier, Ministère de La Santé, Djibouti, Republic of Djibouti; 5 Laboratoire de Diagnostic, Centre de Santé Communautaire d’Einguela, Ministère de La Santé, Djibouti, Republic of Djibouti; 6 Caisse Nationale de Sécurité Sociale (CNSS), Djibouti, Republic of Djibouti; 7 Programme National de Lutte Contre Le Paludisme, Direction des Programmes de Santé Prioritaires, Ministère de La Santé, Djibouti, Republic of Djibouti; 8 Service de Santé des Armées, Présidence de la République, Djibouti, Republic of Djibouti; 9 UNDP Djibouti, Global Fund to Fight AIDS-TB-Malaria, Djibouti, Republic of Djibouti; 10 Unité Parasitologie et Entomologie, Département Microbiologie et Maladies Infectieuses, Institut de Recherche Biomédicale des Armées, Marseille, France

**Correction: Malaria Journal (2023) 22:147** **https://doi.org/10.1186/s12936-023-04546-x**

Following publication of the original article [1], the authors flagged an error in the following sentence: "*Plasmodium falciparum* strains with deleted Pfhrp2 gene, which is less detectable by many currently available RDTs, would then have an advantage over the *P. vivax* strains in the face of control strategies based increasingly on RDTs and post-diagnostic treatment only policy".

Namely, the sentence previously (incorrectly) referred to "‘wild-type’ *P. falciparum* strains" instead of "*P. vivax* strains".

The authors thank you for reading this corrections and apologize for any inconvenience caused.
