# Supplementary material for: Molecular investigation of malaria-infected patients in Djibouti city (2018–2021)
Source: Malar J. 2023 May 3;22:147. doi: 10.1186/s12936-023-04546-x (PMC10154177; doi:10.1186/s12936-023-04546-x)
Supplement: Supplementary file 1 — Additional file 1: Data S1. Number and percentage of positive samples for Plasmodium falciparum and Plasmodium vivax by RDT and PCR in Djibouti city, 2018–2021. [file 12936_2023_4546_MOESM1_ESM.docx]

# Supplementary data 1: Number and percentage of positive samples for *Plasmodium falciparum* and *Plasmodium vivax* by RDT and PCR in Djibouti city, 2018-2021.

|  | **2018** | **2019** | **2020** | **2021^$^** | **TOT** |
| --- | --- | --- | --- | --- | --- |
| ***Plasmodium falciparum*** | | | | | |
| **RDT_Pf+** | 66 | 34 | 68 | 201 | 369 |
| **TOT** | 78 | 196 | 296 | 376 | 946 |
| **%** | 84.6% | 17.3% | 23.0% | 53.5% | 39.0% |
|  |  |  |  |  |  |
| **PCR_Pf+** | 56 | 96 | 237 | 311 | 700 |
| **TOT** | 80 | 187 | 341 | 505 | 1113 |
| **%** | 70.0% | 51.3% | 69.5% | 61.6% | 62.9% |
|  |  |  |  |  |  |
| ***Plasmodium vivax*** | | | | | |
| **RDT_Pv+** | 10 | 31 | 26 | 90 | 157 |
| **TOT** | 78 | 196 | 296 | 376 | 946 |
| **%** | 12.8% | 15.8% | 8.8% | 23.9% | 16.6% |
|  |  |  |  |  |  |
| **PCR_Pv+** | 15 | 36 | 33 | 48 | 132 |
| **TOT** | 80 | 187 | 341 | 505 | 1113 |
| **%** | 18.8% | 19.3% | 9.7% | 9.5% | 11.9% |
|  |  |  |  |  |  |
| **Pan plasmodium** | |  |  |  |  |
| **nPf+/n Pv+** | 3.73 | 2.67 | 7.18 | 6.48 | 5.30 |
|  |  |  |  |  |  |
| **Infected** | 65 | 120 | 259 | 344 | 788 |
| **TOT** | 80 | 187 | 341 | 505 | 1113 |
| **%** | 81.2% | 64.2% | 75.9% | 68.1% | 70.8% |

^$^In 2018–2020, CareStart® malaria Pf/Pv HRP2/pLDH RDT was used. In 2021, Biosynex® malaria P.f/pan pLDH was used. %: percentage of samples infected. Pf+: samples infected by *Plasmodium falciparum* (mixed Pf/Pv infections included). Pv+: samples infected by *Plasmodium vivax* (mixed Pf/Pv infections included). nPf+/n Pv+ : Ratio of *falciparum* to *vivax* plasmodial species detected by PCR. Infected: Total number of plasmodial infections detected by PCR - mixed Pf/Pv infections are counted only once
