# Supplementary material for: Molecular investigation of malaria-infected patients in Djibouti city (2018–2021)
Source: Malar J. 2023 May 3;22:147. doi: 10.1186/s12936-023-04546-x (PMC10154177; doi:10.1186/s12936-023-04546-x)
Supplement: Supplementary file 2 — Additional file 2: Figure S1. Age of patients by malaria status from four health facilities in Djibouti city between 2019 and 2021. [file 12936_2023_4546_MOESM2_ESM.docx]

# Supplementary fig 1: Age of patients by malaria status from four health facilities in Djibouti city between 2019 and 2021.

The age distribution of the patients is represented by decades from 0 to 9 years, 10 to 19 years, up to 99 years. Malaria status is based on PCR result, plasmodial infection (red) or negative (blue). The general Djiboutian population, according to the updated General Census of Population and Housing, is represented by a grey dotted line. The proportion of infected and uninfected patients differed significantly in the age groups 0-9 years (*P*=0.04), 10-29 (*P*=0.004) and 60-79 years (*P*=0.02).
